# *Lactobacillus paracasei* from koumiss mediates intestinal stem cells to promote intestinal mucosal recovery through intestinal microbiota-metabolites

**DOI:** 10.3389/fnut.2026.1893728

**Published:** 2026-08-26

**Authors:** Shunan Ren, Jincheng Li, Qi Zhang, Ying Han, Guifang Cao

**Affiliations:** College of Veterinary Medicine, Inner Mongolia Agricultural University, Hohhot, China

**Keywords:** FMT, gut microbiota, intestinal stem cell, mucosal repair, probiotic

## Abstract

**Background:**

Infectious diarrhea caused by *Salmonella typhimurium* (*S. typhimurium*) leads to intestinal barrier dysfunction, dysbiosis, and impaired epithelial regeneration. Current antibiotic therapies are prone to adverse effects such as drug resistance and drug residues, whereas probiotic interventions have shown beneficial effects. In this context, the study focused on a traditional fermented product called koumiss, from which a strain of *Lactobacillus paracasei* (*L. paracasei*) was isolated. Previous studies have demonstrated that this strain is capable of repairing the damaged intestinal mucosal barrier. However, its underlying mechanism of action remains unclear.

**Methods:**

Seventy Kunming mice were randomly divided into control group, model group, *L. paracasei* intervention group, and *Lactobacillus plantarum* (*L. plantarum*) intervention group. Except for the control group, the diarrhea model induced by *S. typhimurium* was established in the other groups. Serum inflammatory factors, intestinal permeability, and immune indexes were detected by enzyme-linked immunosorbent assay. Hematoxylin eosin, Alcian blue-periodic acid–Schiff, electron microscopy, immunofluorescence, reverse transcription quantitative polymerase chain reaction, and Western blot were used to evaluate the intestinal barrier function and the ability of intestinal stem cells to proliferate and differentiate into Paneth cells and goblet cells. The structure of the intestinal flora and the content of short-chain fatty acids (SCFAs) were detected by 16S ribosomal ribonucleic acid, and gas chromatography–mass spectrometry. Furthermore, fecal microbiota transplantation was performed by transferring feces from *L. paracasei*-intervened mice to germ-free mice, in order to determine whether the microbiota reshaped by *L. paracasei* could independently mediate intestinal repair and to verify the interaction between the microbiota and metabolites.

**Results:**

*L. paracasei* and *L. plantarum* can significantly reduce diarrhea symptoms, weight loss, and intestinal barrier damage, reduced serum lipopolysaccharide, diamine oxide, D-lactate, and zonulin levels, promote the expression of tight junction proteins, and promote the proliferation and differentiation of Lgr5^+^ intestinal stem cells, increased the expression of Paneth cells (lysozyme, Defa5, Ang4) and goblet cells (MUC2, TFF3) markers, and enhanced the protective effect of the physical mucus barrier on the intestine, but the protective effect of *L. paracasei* on the intestinal mucosa is better than that of *L. plantarum*. 16S rRNA sequencing showed that compared with *L. plantarum*, *L. paracasei* intervention enriched the Muribaculaceae and Prevotellaceae families that produced SCFAs. The results of fecal microbiota transplantation showed that the transplantation of *L. paracasei* in the feces of mice still had the protective effect of intestinal mucosal barrier, which could restore the expression of intestinal stem cells, improve the intestinal mucosal barrier, reduce inflammation and increase the level of SCFAs, indicating that *L. paracasei* mediated the repair of intestinal mucosal barrier damage through intestinal microbiota-metabolites.

**Conclusion:**

Both *L. paracasei* and *L. plantarum* are capable of repairing intestinal injury, however, *L. paracasei* is proved better than *L. plantarum*. *L. paracasei* ameliorates infectious diarrhea by remodeling the gut microbiota, enhancing production of SCFAs, suppressing inflammation, preserving Paneth cell niche function, and ultimately promoting Lgr5^+^ intestinal epithelium and stem cell (ISC)-mediated epithelial regeneration. These findings provide a mechanistic basis for probiotic intervention in infectious diarrhea and highlight the microbiota–ISC axis as a therapeutic target.

## Introduction

The intestinal mucosal barrier serves as the first line of defense against pathogenic microbial invasion and the maintenance of internal environment homeostasis. Its integrity relies on the synergistic action of tight junctions of epithelial cells, the mucus layer, and innate immune cells (1). When the intestinal barrier is compromised, pathogens and toxins (such as lipopolysaccharide) can cross the intestinal epithelium and enter the circulatory system, inducing local and even systemic inflammatory responses (2–4). Diarrhea is one of the most common gastrointestinal disorders in humans and animals, characterized by impaired intestinal barrier function, excessive activation of inflammatory responses, gut microbiota dysbiosis, and reduced regenerative capacity of the intestinal epithelium (3, 5, 6). Among these, infectious diarrhea caused by enteric pathogens such as *Salmonella typhimurium* can lead to necrosis of intestinal epithelial cells, villus atrophy, disruption of crypt structure, and dysfunction of goblet cells and Paneth cells, thereby weakening the physical and chemical defense capabilities of the intestine (7, 8).

Probiotics are live microorganisms when administered in adequate amounts, confer health benefits to the host (9–11). Specific lactic acid bacteria can alleviate intestinal inflammatory damage, maintain homeostasis of the gut microbiota, enhance intestinal barrier integrity, and promote mucosal repair (12–14). *L. paracasei* is a facultatively heterofermentative lactic acid bacterium that has garnered widespread attention due to its desirable properties, including tolerance to gastrointestinal stress, strong intestinal colonization ability, modulation of gut microbiota structure, anti-inflammatory activity, and enhancement of mucosal immunity (15). Recent studies have further advanced the current understanding and role that *L. paracasei* play in intestinal health. For instance, *Lactobacillus paracasei* E10 was shown to alleviate DSS-induced colitis by enhancing intestinal barrier integrity, increasing goblet cell density, and enriching the short-chain fatty acids (SCFAs)-producing family Muribaculaceae (16). The postbiotic of *Lactobacillus paracasei* JY062 exerted multi-target therapeutic effects on colitis through integrated antioxidant, barrier repair, immunomodulatory, and microbiota modulation, including restoration of intestinal barrier integrity and elevation of fecal SCFA levels (17). However, the precise molecular mechanisms by which *L. paracasei* antagonizes pathogenic infection, regulates intestinal stem cell function, and promotes intestinal mucosal repair remain to be fully elucidated.

The continuous renewal and injury repair of the intestinal epithelium depend on intestinal stem cells, which are located at the base of the crypts and can differentiate into various functional cell types, including goblet cells, Paneth cells, absorptive epithelial cells, and enteroendocrine cells (18, 19). Goblet cells secrete mucin MUC2 to form the mucus barrier, whereas Paneth cells produce antimicrobial substances such as lysozyme, defensin Defa5, and angiogenin Ang4 to maintain intestinal innate immune homeostasis. Impairment of intestinal stem cell activity and epithelial differentiation function directly leads to defective mucosal repair and persistent intestinal injury. Furthermore, the gut microbiota and its metabolites, particularly SCFAs, play an irreplaceable role in regulating intestinal stem cell function, epithelial proliferation, and inflammatory responses. Emerging evidence has established that the gut microbiota and its metabolites play an irreplaceable role in regulating ISC function, epithelial proliferation, and inflammatory responses. SCFAs such as acetate, propionate, and butyrate not only serve as important energy sources for colonic epithelial cells but also act as key signaling molecules that modulate immune homeostasis, barrier function, and microbial balance (20, 21).

In this study, a mouse model of infectious diarrhea induced by *S. typhimurium* was established to systematically investigate the protective effects of *L. paracasei* and *Lactobacillus plantarum* against intestinal mucosal injury, and to evaluate their impacts on the intestinal barrier, inflammatory responses, intestinal tissue morphology, epithelial regeneration, and gut microbiota structure.

## Materials and methods

### Ethic statement

All animal experiments were conducted according to the ethical policies and procedures approved by the Animal Care and Use Committee of Inner Mongolia Agricultural University (no. NND2026161).

### Bacterial strains

*L. plantarum* and *L. paracasei* strains used in this study was isolated from koumiss and stored at −80 °C. The strain was sub-cultured three times in de Man, Rogosa, and Sharpe (MRS) media at 37 °C for 48 h after thawing. The colony counting method was used to adjust the *L. paracasei* and *L. plantarum* concentration to 3 × 10^8^ CFU/mL. *S. typhimurium* was purchased from BNCC (strain CMCCB50222), the strain was prepared from 12 h shake culture grown and adjusted to a concentration of 10^8^ CFU/mL.

### Animal experiments

**Figure 1:**
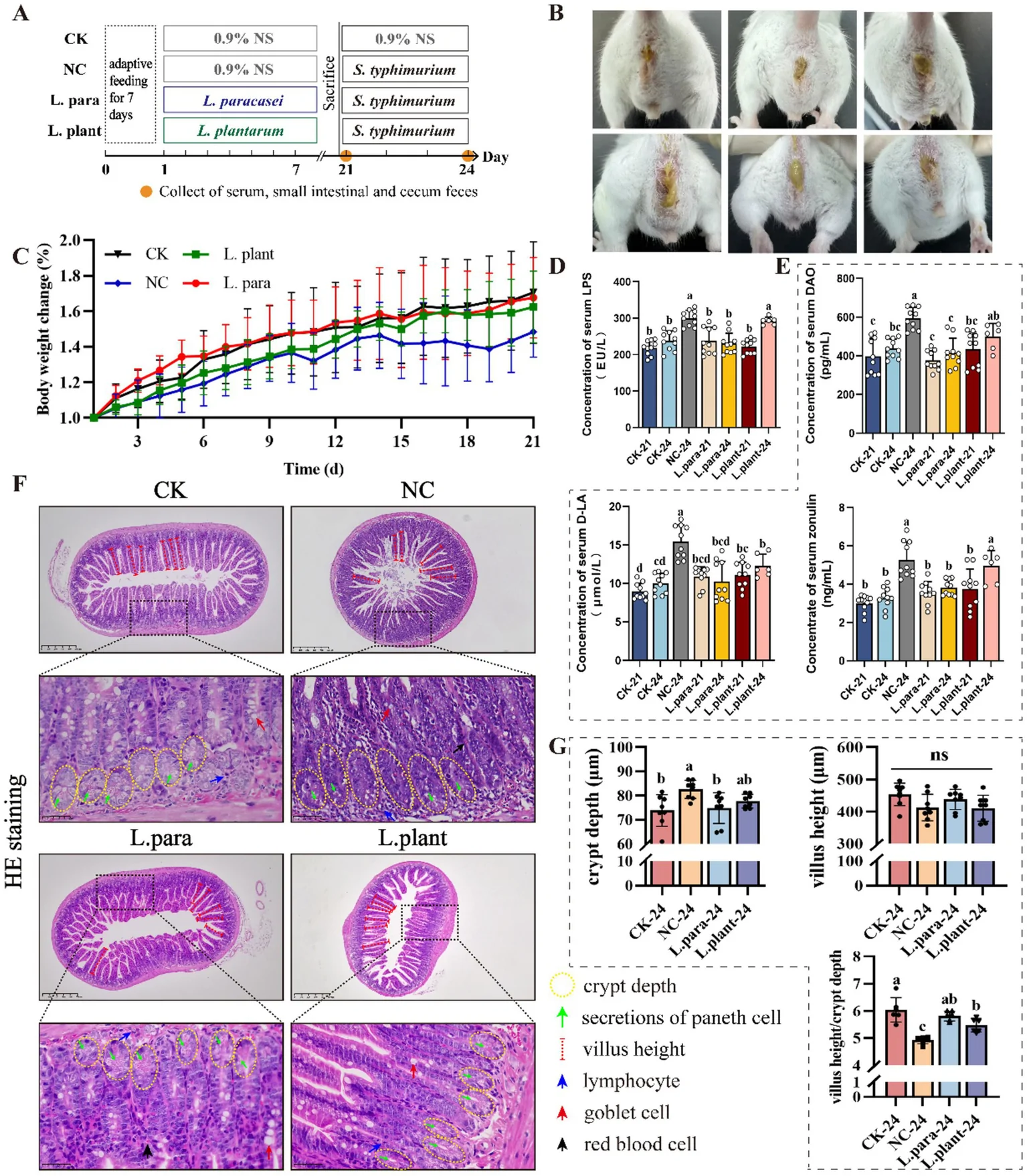
*L. paracasei* and *L. plantarum* prevent damage to the intestinal mucosa caused by *S. typhimurium*. **(A)** Experimental scheme (day 21 CK, *L. paracasei* and *L. plantarum* sampling *n* = 10; day 24 CK, NC, *L. paracasei* and *L. plantarum* sampling *n* = 10). **(B)** Mice in the NC group developed diarrhea symptoms after intervention with *S. typhimurium*, including perianal stool staining and representative photographs of hair stains. **(C)** The body weight change in *S. typhimurium* treated mice supplemented with *L. paracasei* and *L. plantarum* individually. **(D)** The content of serum LPS. **(E)** The content of serum DAO, D-LA, and zonulin. **(F)** H&E staining of the ileum in *S. typhimurium* treated mice supplemented with *L. paracasei* and *L. plantarum* individually. **(G)** The crypt depth, villus height, and the ratio of villus height to crypt depth. One-way ANOVA with Tukey’s test was used for statistical analysis of all parameters. Data were presented as mean ± SEM. Different lowercase letters indicate significant differences between groups (*p* < 0.05); the same letter indicates no significant difference (*p* > 0.05).

Seventy specific-pathogen-free Kunming mice (age, 3–4 weeks; weight, 18–22 g) were obtained from the Inner Mongolia Medical University Experimental Animal Center, Hohhot, China. The mice were raised under specific pathogen-free conditions with a 12 h light and 12 h dark cycle at 20 °C ± 2 °C, maintained under 45% ± 10% humidity, and had *ad libitum* access to sterilized food and water. After a week of acclimatization, the mice were randomly divided into four groups (Figure 1A): control (CK) (*n* = 20), model control (NC) (*n* = 10), *L. paracasei* group (*L. para*) (*n* = 20), and *L. plantarum* (*L. plant*) group (*n* = 20). The small sample size of the NC group (*n* = 10) was due to the fact that the group did not receive probiotic intervention, did not sample on the 21st day, and only sampled tissues at the end of the experiment on day 24. In contrast, the other groups required 10 mice for sampling on day 21 and another 10 for sampling on day 24. This design minimizes the use of animals according to the 3R principle. No animals were lost during the study. The detailed rules for each group are as follows: The control group received daily oral gavage 0.2 mL/10 g of 0.9% saline for 24 days. The diarrhea model control group was gavaged with 0.2 mL/10 g of 0.9% saline for the first 21 days, followed by 3-day administration of *S. typhimurium* (*S. typh*) to establish the infectious diarrhea model. The *L. paracasei* and *L. plantarum* groups were administered their respective bacterial suspensions daily for 21 days, followed by the same 3 days of *S. typhimurium* challenge. Samples were collected on 21 d for serum and cecum contents from all mice, and on 24 d serum, cecal contents, and ileum tissues were collected.

For fecal microbiota transplantation (FMT), a cocktail of antibiotics (25 mg/kg vancomycin, 50 mg/kg ampicillin, 50 mg/kg neomycin sulfate, 50 mg/kg metronidazole), were gavaged for 2 weeks to deplete the intestinal microbiota. Mice were then orally administered with 0.2 mL phosphate-buffered saline (PBS) or fecal suspension daily for 2 weeks. The fecal suspension derived from the mice administered with *L. paracasei*, followed by suspension of 100 mg feces in 1 mL sterile anaerobic PBS. Prior to sampling, mice were fasted for 12 h. Ileum tissues were collected from each group. Tissue samples were gently rinsed with PBS, fixed in 4% paraformaldehyde, and subsequently used for H&E staining, AB-PAS staining, immunohistochemistry, immunofluorescence, and other assays.

### Histopathological sections

Fixed ileum tissues were rinsed under running water for 12 h, dehydrated through a graded ethanol series (70% ethanol for 40 min, 80% ethanol for 40 min, 90% ethanol for 1 h, 95% ethanol twice for 30 min each, absolute ethanol twice for 25 min each), treated with a 1:1 mixture of ethanol and xylene for 10 min, cleared in xylene (twice for 20 min each), infiltrated with paraffin for 1 h, and embedded in paraffin blocks. The embedded tissue blocks were sectioned at a thickness of 4–5 μm. Sections were floated in 40 °C distilled water for mounting and dried on a slide dryer at 37 °C. For hematoxylin and eosin staining paraffin sections were deparaffinized in xylene, treated with 1:1 mixture of xylene and absolute ethanol for 3 min, rehydrated through a graded ethanol series (absolute ethanol, 90% ethanol, 70% ethanol for 3 min each), stained with hematoxylin for 10 min, differentiated in acid alcohol for 5 s, blued in lithium carbonate for 5 s, rinsed under running water for 15 min, and counterstained with eosin for 2 min. Sections were then dehydrated through graded ethanol (70% ethanol, 90% ethanol, absolute ethanol for 3 min each), cleared in xylene, mounted with neutral balsam, and examined under a microscope. For AB-PAS staining paraffin sections were deparaffinized in xylene and washed with distilled water for 2 min. Alcian blue staining solution was applied for 10–20 min, followed by three washes with distilled water. Sections were immersed in oxidizer for 5–8 min, washed twice with distilled water. Schiff’s staining solution was applied to cover the sections for 10–20 min, followed by two washes with distilled water (5 min each). Nuclei were counterstained with hematoxylin for 1–2 min and rinsed with distilled water. Differentiation was performed with acid alcohol for 2–5 s, followed by a distilled water rinse. Sections were blued in Scott’s tap water substitute for 3 min and washed with distilled water for 3 min. Finally, sections were dehydrated through graded ethanol (70% ethanol, 90% ethanol, absolute ethanol for 3 min each), cleared in xylene, mounted with neutral balsam, and examined under a microscope.

### Immunofluorescence staining

Paraffin sections were baked at 60 °C for 1 h, deparaffinized in xylene (three times, 10 min each), and rehydrated through graded ethanol (absolute ethanol three times for 3 min each, 95% ethanol three times for 2 min each, 75% ethanol three times for 2 min each), followed by a 1 min wash in distilled water. Antigen retrieval was performed by heating the sections in citrate buffer (pH 6.0) in a microwave for 15 min, followed by natural cooling. Endogenous peroxidase was blocked by incubating with blocking solution at room temperature for 10 min, followed by PBS washes (three times for 3 min each). Sections were incubated with MUC2 (HUABIO, ET1704-06, 1:500), Lgr5 (affinity, DF2816, 1:100), lysozyme (abcam, AB108508, 1:200) overnight at 4 °C, washed with PBS (three times for 3 min each), and incubated with fluorescence-labeled secondary antibody at room temperature for 2 h in the dark. After PBS washes (three times for 3 min each), nuclei were stained with DAPI for 10 min in the dark, followed by PBS washes (three times for 3 min each). Sections were mounted with anti-fluorescence quenching mounting medium and examined under a fluorescence microscope.

### Enzyme-linked immunosorbent assay

During the study, mice blood samples were drawn into red top vacuum tubes and incubated at 37 °C for 2 h, followed by overnight storage at 4 °C to allow complete clot retraction. After natural precipitation, the samples were centrifuged at 3,000 rpm for 10 min at 4 °C, and the sera were aliquoted and stored at −80 °C until further analysis. Serum levels of intestinal permeability markers, including diamine oxidase (DAO), D-lactate (D-LA), and zonulin, as well as immunoglobulins (IgM, IgG, and IgA) and inflammatory cytokines, including pro-inflammatory cytokines (IL-6, IL-1β, and TNF-*α*) and the anti-inflammatory cytokine IL-10, were determined using corresponding rat enzyme-linked immunosorbent assay (ELISA) kits (Elabscience, Bethesda, MD, USA) following the manufacturer’s protocols.

For tissue sample preparation, ileal tissues were collected and homogenized in lysis buffer (1.8 mL per 0.2 g tissue) containing 50 mM Tris–HCl (pH 7.4), 150 mM NaCl, 1% Triton X-100, 1% sodium deoxycholate, 0.1% SDS, sodium orthovanadate, sodium fluoride, EDTA, and leptin. The homogenates were centrifuged at 5,000 rpm for 10 min at 4 °C, and the resulting supernatants were collected and stored at −80 °C. The secretory immunoglobulin A (sIgA) levels in tissue homogenates were measured using a rat sIgA ELISA kit (Elabscience, Bethesda, MD, USA). All ELISA procedures were strictly carried out in accordance with the manufacturers’ instructions, and each sample was assayed in triplicate to ensure reproducibility.

### RNA extraction, reverse transcription, and RT-qPCR

Total RNA was extracted from tissues using the RNA Extraction Kit (UElandy, China) according to the manufacturer’s instructions. Reverse transcription was performed using the HiScript II Q RT SuperMix for qPCR (Vazyme, China) to synthesize cDNA. Using cDNA as a template, real-time quantitative PCR was performed with the ChamQ Universal SYBR qPCR Master Mix (Vazyme, China) on a real-time PCR system. Primers were synthesized by Sangon Biotech (Shanghai) Co., Ltd. (China), shown in Table 1. *β*-Actin was used as the reference gene, and the relative expression levels of target genes were calculated using the 2^-ΔΔCt^ method.

**Table 1:** Primer sequences used in RT-qPCR.

Genes	Forward sequence (5′–3′)	Reverse sequence (5′–3′)
Defa5	CCCCCAGCCATGAAGACATT	GACACAGCCTGGTCCTCTTC
Ang4	GCTAACCTCGCCTTGCAAAG	CCTGAGTGCGTACAAGTGGT
TFF3	TGCAGATTACGTTGGCCTGT	ACACTGCTCCGATGTGACAG

### Protein extraction, quantification, and western blot

Total protein was extracted from ileum tissues using the IntactProtein^™^ Universal Protein Extraction Kit (#415, Shanben Biology, China). Extracted proteins were quantified with the BCA method. Protein samples were separated by SDS-PAGE gel (Omni-Easy^™^ One-Step PAGE Gel Rapid Preparation Kit, EpiZyme, China) using SDS-PAGE running buffer (P0014A, Beyotime, China) and transferred to membranes via semi-dry transfer using Western Semi-Dry Transfer Buffer (P0021C, Beyotime, China). After transfer, membranes were blocked with 5% non-fat milk at room temperature for 1 h and incubated with primary antibodies overnight at 4 °C. Western blot was performed with the following antibodies: *β*-actin (Affinity, AF7018, 1:10000), Occludin (HUABIO, ET1701-76, 1:1000), and HRP-conjugated secondary (Affinity, S0001, 1:5000). After stripes collection, the signals were quantified using Image J software.

### DNA extraction and 16S rRNA gene sequencing and analysis

Total microbial genomic DNA was extracted from fecal samples using the E.Z.N.A.^®^ soil DNA Kit (Omega Bio-tek, Norcross, GA, United States) according to manufacturer’s instructions. The hypervariable region V3-V4 of the bacterial 16S rRNA gene were amplified with primer pairs 338F/806R on Illumina Nextseq2000 platform. Data analysis was performed on the free online platform of majorbio Cloud Platform.^1^ Principal coordinates analysis (PCoA) was based on unweighted Unifrac distances. Differentially enriched bacterial taxa were based on Kruskal–Wallis test and post-hoc Dunn’s test.

### SCFAs detection

Preparation of mixed standard solution. Take 9,840 μL of HPLC grade n-butanol, put it into a 15 mL centrifuge tube, add appropriate amounts of acetic acid, propanoic acid, butanoic acid, isobutyric acid, valeric acid, isovaleric acid, hexanoic acid, and isohexanoic acid in turn. Vortex and mix well to obtain the mixed standard stock solution A for eight SCFAs. Preparation of internal standard: Take 9,990 μL of HPLC grade n-butanol into a 15 mL centrifuge tube, add 10 μL of internal standard 2-ethylbutyric acid, vortex and mix well to obtain the internal standard stock solution B. Then the above mixed standard A and B solutions were diluted with n-butanol into seven different concentrations of working solutions and loaded into the injection vials for GC–MS detection and analysis. A measure of 25 mg feces sample was accurately weighed into 2 mL grinding tube and 500 μL of water added containing 0.5% phosphoric acid. The samples were frozen and ground at 50 Hz for 3 min, and the process was repeated twice, followed by ultrasound for 10 min and centrifugation at 4 °C and 13,000 g for 15 min. A volume of 200 μL of the supernatant was aliquoted into a 1.5 mL centrifuge tube, and 0.2 mL of N-butanol solvent containing internal standard 2-ethylbutyric acid (10 μg/mL) was added for exaction. Finally, the solution was vortexed for 10 s, followed by ultrasound at low temperature for 10 min, and then centrifuged at 4 °C and 13,000 g for 5 min and the supernatant was carefully transferred to sample vials for analysis. The analysis was performed using an Agilent 8890B gas chromatography coupled with an Agilent 5977B/7000D mass selective detector with an inert electron impact ionization source at an ionization voltage of 70 eV (Agilent, USA) at Majorbio Bio-Pharm Technology Co. Ltd. (Shanghai, China). The GC–MS raw data were imported into Masshunter quantitative software (Agilent, USA, version number: v10.0.707.0). All ion fragments were automatically identified and integrated by using default parameters, besides, all integration was checked manually. The metabolite concentration of samples was calculated according to linear regression standard curve.

## Results

### *Lactobacillus paracasei* and *Lactobacillus plantarum* have the effect of improving intestinal barrier dysfunction

To elucidate the protective effects of *L. paracasei* and *L. plantarum* on the intestinal mucosal barrier, this study was conducted using a mouse model of diarrhea. After administration of *S. typhimurium*, the mice developed diarrhea (Figure 1B). Both *L. paracasei* and *L. plantarum* significantly attenuated body weight loss and reduced serum levels of lipopolysaccharide (LPS), diamine oxidase (DAO), D-lactate (D-LA), and zonulin (Figures 1C–E), indicating that both strains alleviated intestinal barrier dysfunction. However, *L. paracasei* consistently showed a greater effect than *L. plantarum* on these parameters. Compared with CK, NC group oral administration with *S. typhimurium* significantly increased the serum content of LPS (Figure 1D, *P*<0.05), while the intestinal permeability indicators, DAO, D-LA, and zonulin were measured by ELISA, the result showed that *S. typhimurium* increased the content of DAO and D-LA (Figure 1E, *P*<0.05), indicated that *S. typhimurium* induced intestinal permeability and damaged the epithelium of the intestinal mucosa. Histopathological observation of the ileum revealed that villi became shorter after *S. typhimurium* treatment (Figure 1F), but there was no significance between NC and other groups (*P*>0.05), however, the crypt depth was significantly increased in the NC group (*P*<0.05), and the ratio of villus height to crypt depth (V/C) was calculated, showing that *S. typhimurium* administration reduced the V/C of ileum tissues (Figure 1G). Normal intestinal tissue exhibits structurally intact mucosal epithelium with sparse lymphocyte infiltration. However, following *S. typhimurium* challenge, the intestinal mucosa shows extensive epithelial sloughing and necrosis, accompanied by marked inflammatory infiltration in the submucosal layer. Additionally, crypt structures display vascular dilation and lymphocyte infiltration, along with reduced secretory activity of Paneth cells. *L. paracasei* and *L. plantarum* maintained normal mucosal morphology without inducing intestinal damage. Notably, *L. paracasei* was more effective than *L. plantarum* in restoring villus architecture and reducing crypt depth. Supporting that *L. paracasei* has a better effect on protective intestinal mucosal barrier integrity.

### *Lactobacillus paracasei* and *Lactobacillus plantarum* enhance tight junction integrity by reducing inflammatory responses

**Figure 2:**
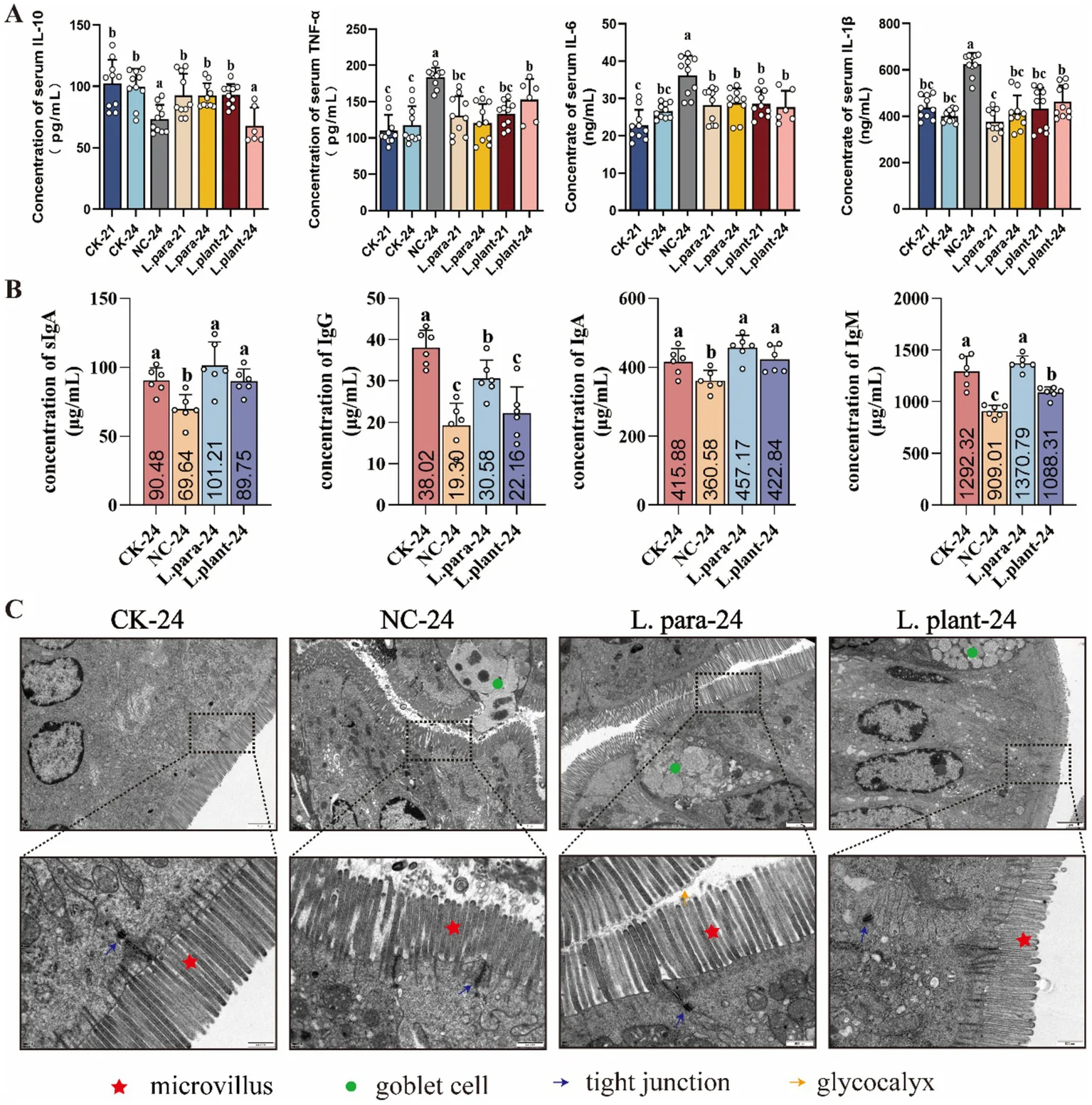
*L. paracasei* and *L. plantarum* alleviate inflammation and promote immunoglobulin secretion. **(A)** Serum levels of the anti-inflammatory cytokine IL-10 and the pro-inflammatory cytokines TNF-*α*, IL-6, and IL-1β. **(B)** The levels of IgA, IgG, IgM, and sIgA. **(C)** The ultrastructure of ileal tissue. One-way ANOVA with Tukey’s test was used for statistical analysis of all parameters. Data were presented as mean ± SEM. Different lowercase letters indicate significant differences between groups (p < 0.05); the same letter indicates no significant difference (*p* > 0.05).

To explain the effects of *L. paracasei* on the expression of intestinal pro-inflammatory and anti-inflammatory factors, the levels of IL-10, TNF-*α*, IL-1β, and IL-6 in serum were measured (Figure 2A). Additionally, the concentrations of serum immunoglobulins (IgM, IgG, and IgA) and the level of secretory IgA (sIgA) in intestinal tissue homogenates were determined (Figure 2B). The results showed the serum content of TNF-*α*, IL-6, and IL-1β significant increase in NC group, but both probiotic interventions significantly reduced those pro-inflammatory cytokines expression, while elevating the anti-inflammatory cytokine IL-10, immunoglobulin levels (IgA, IgG, IgM) and the expression of sIgA in ileal tissue homogenate (Figures 2A,B). Notably, *L. paracasei* elicited a more pronounced suppression of TNF-α and IL-1β and a stronger elevation of IL-10 compared with *L. plantarum*. Subsequently, electron microscopy was used to observe the ultrastructure of the ileal tissue (Figure 2C). In the NC group, the ileal microvilli were more severely disrupted compared to the other groups. In contrast, the *L. paracasei* group exhibited more distinct glycocalyx on the surface of the microvilli, along with relatively intact tight junctions, whereas *L. paracasei* showed only moderate improvement. These results suggest that *L. paracasei* is more effective than *L. plantarum* in suppressing systemic inflammation and preserving the ultrastructural integrity of the intestinal epithelium.

### *Lactobacillus paracasei* mediate intestinal epithelial cell proliferation and differentiation via alterations in gut microbiota composition

**Figure 3:**
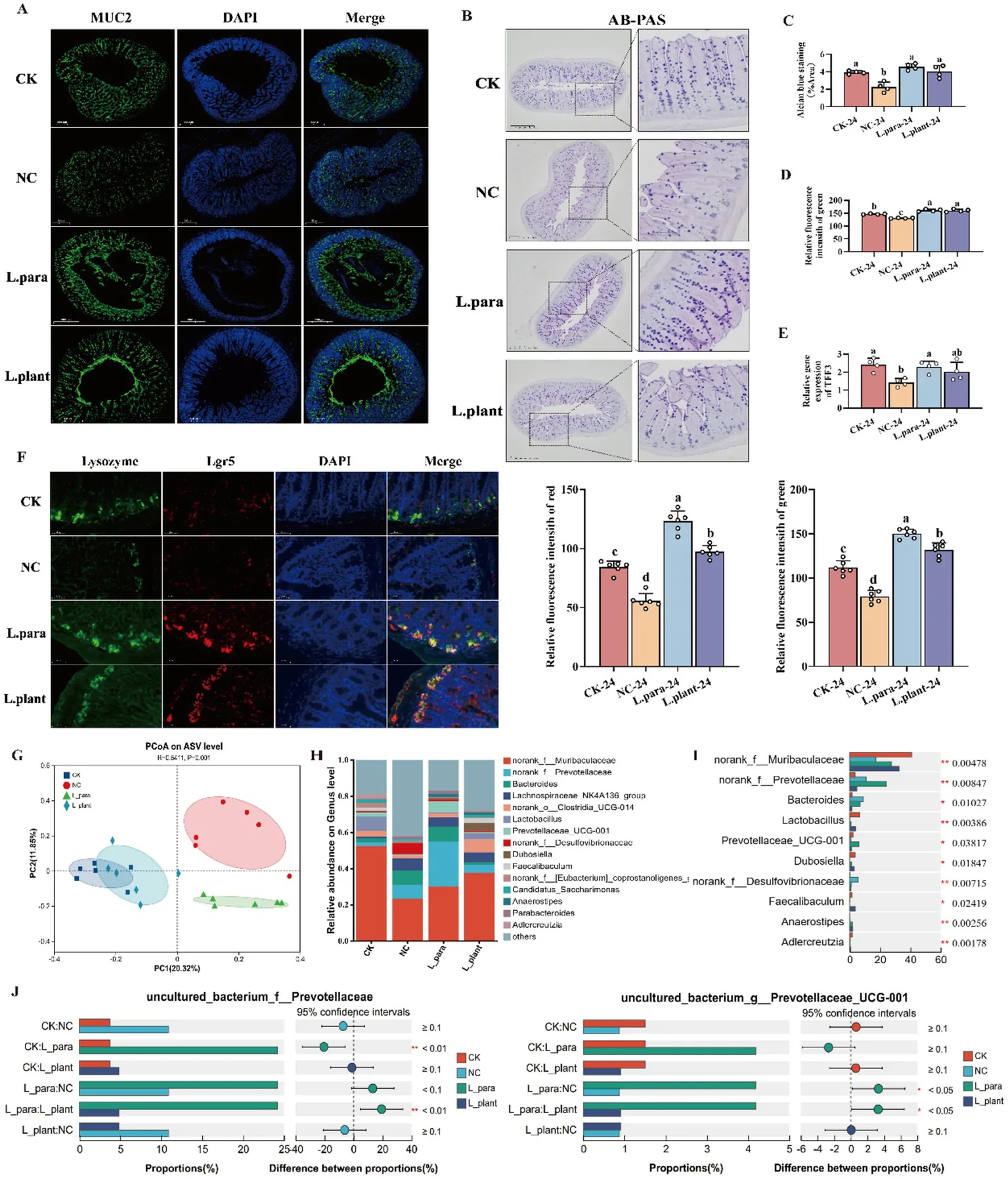
*Lactobacillus paracasei* and *Lactobacillus plantarum* promote the proliferation and differentiation of intestinal stem cells to repair damaged intestinal epithelium by changing the composition of intestinal flora. **(A,D)** Ileum MUC2 immunofluorescence, and intensive. **(B,C)** Ileum AB-PAS staining, and histological damage score of the ileum in *S. typhimurium*-treated mice supplemented with *L. paracasei* and *L. plantarum*. **(E)** Representative expression of TFF3 in the ileum at day 24 after *L. paracasei*, *L. plantarum*, and *S. typhimurium* administered using RT-qPCR in mice. **(F)** Representative photomicrographs of immunofluorescence staining of ileal lysozyme and Lgr5 on day 24. **(G)** Principal coordinate analysis (PCoA) of the gut microbiome composition at the operational taxonomic unit (OUT) level based on the weighted_UniFrac matrix for each group. The shapes and colors of the spots indicate samples from each individual. Colored ellipses indicate 0.95 confidence interval ranges within each tested group. *N* = 6 mice per group. **(H)** Relative abundance of the gut bacterial genus level in each group. *N* = 6 mice per group. **(I)** The fecal microbiota at the species levels. **(J)** Differences between groups of Prevotellaceae. One-way ANOVA with Tukey’s test was used for statistical analysis. Data were presented as mean ± SEM. Different lowercase letters indicate significant differences between groups (*p* < 0.05); the same letter indicates no significant difference (*p* > 0.05).

To investigate the ability of *L. paracasei* and *L. plantarum* to repair intestinal injury, the expression of cell type specific markers was analyzed in the intestinal epithelium. Specifically, the goblet cell marker MUC2 and the Paneth cell marker lysozyme were measured. Additionally, given that epithelial renewal depends on intestinal stem cells, the expression of the stem cell marker Lgr5 was quantified. Figure 3A shows MUC2 expression significantly increases in *L. paracasei* and *L. plantarum* compared with the negative group (Figure 3D). Meanwhile, AB-PAS staining was used (Figure 3B) to assess mucin expression under different interventions. The results showed that mucin expression in the NC group was significantly lower than that in all other groups, while no significant differences in mucin expression were observed between the *L. paracasei* and *L. plantarum* groups compared with the CK group (Figure 3C). The expression of lysozyme and Lgr5 was significantly reduced in the NC group, indicating that diarrhea led to loss of stem cell renewal function. However, following intervention with *L. paracasei* and *L. plantarum*, the expression of Lgr5 and Paneth cells was increased (Figure 3F), suggesting that probiotics promote the proliferation and differentiation of intestinal stem cells and enhance the intestinal ability to defend against pathogenic microorganisms. Given that Paneth cells secrete antimicrobial peptides and other substances, the mRNA expression level of TFF3 using RT-qPCR was further examined. Similarly, TFF3 expression was significantly lower in the model group than in all other groups (Figure 3E). These results indicate that probiotics, particularly *L. paracasei* promote intestinal epithelial cell regeneration and protect against pathogen-induced intestinal damage.

The probiotics rejuvenate the intestinal epithelium by entering the intestines and changing the composition of the intestinal microbiota, therefore, 16S rRNA sequencing was performed. While *S. typhimurium* caused an obvious shift in the ileum microbiota composition, the probiotic led to further alterations without restoring it back to normal (Figure 3G). Both probiotic treatments altered the overall microbial composition, *L. paracasei* significantly enriched the SCFA-producing families Muribaculaceae and Prevotellaceae, which was drastically reduced in the NC group (Figures 3H,I). In contrast, *L. plantarum* failed to restore Prevotellaceae abundance. Furthermore, although *L. plantarum* has been reported to enrich Prevotellaceae, the study did not observe such enrichment in the *S. typhimurium*-infected model (Figure 3J). These results suggest that the specific enrichment of Muribaculaceae and Prevotellaceae by *L. paracasei* may underlie its superior ability to promote ISC-mediated epithelial repair.

### Role of *Lactobacillus paracasei* derived microbiota via FMT in intestinal repair of a mouse model with intestinal injury

**Figure 4:**
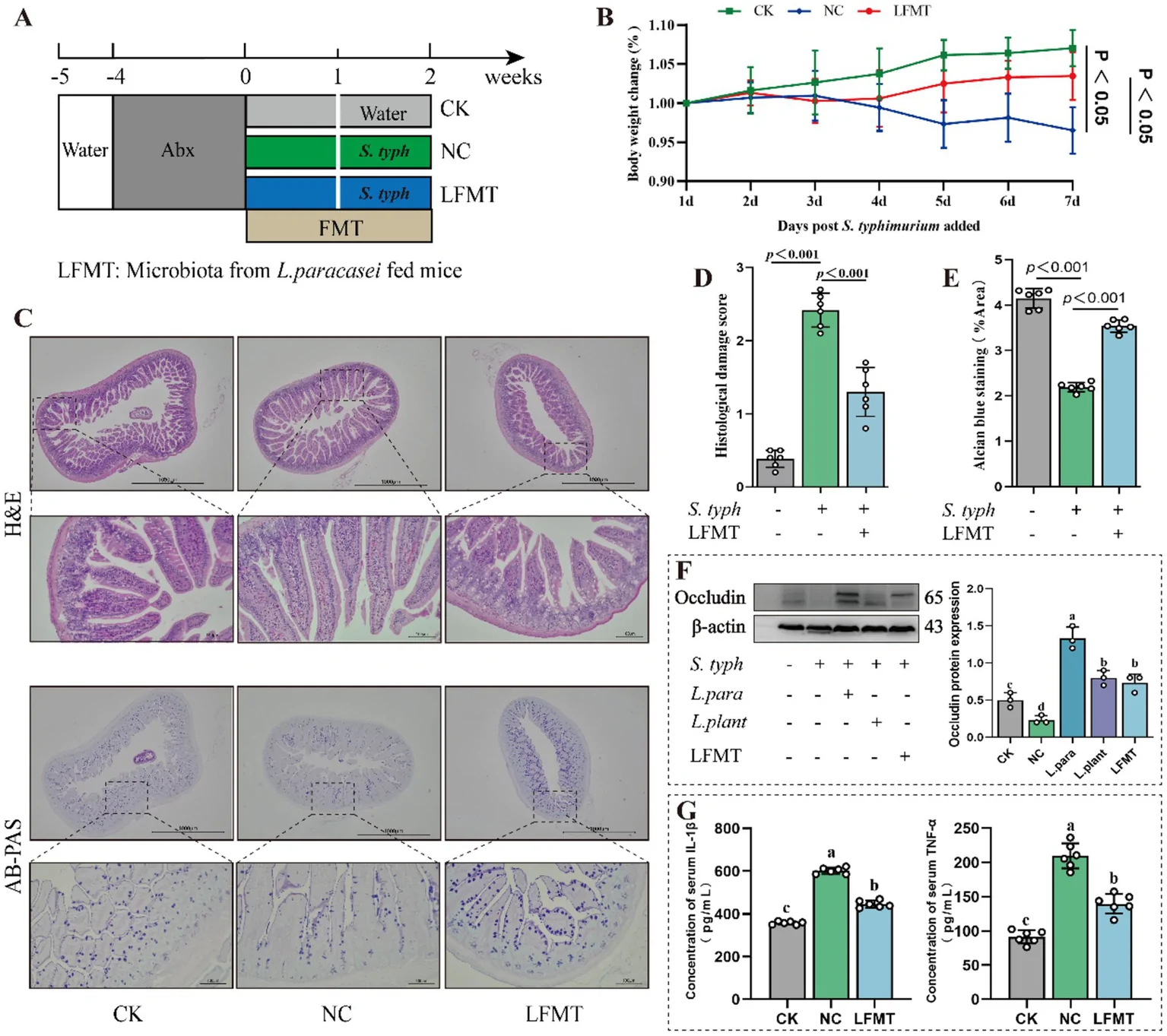
Intestinal mucosal injury repaired in *S. typhimurium*-treated mice transplanted with the fecal microbiota of *L. paracasei*-supplemented mice. **(A,B)** Study design and body weight change in *S. typhimurium*-treated mice with/without treatment with LFMT. **(C–E)** The H&E staining, Alcian blue staining, histological damage score of the ileal of *S. typhimurium*-treated mice treatment with LFMT. **(F,G)** Relative protein expression levels of tight junction proteins and serum concentrations of pro-inflammatory cytokines in the ilea of *S. typhimurium*-treated mice with LFMT. One-way ANOVA with Tukey’s test was used for statistical analysis. Data were presented as mean ± SEM. Different lowercase letters indicate significant differences between groups (*p* < 0.05); the same letter indicates no significant difference (*p* > 0.05).

Given the superior protective effects of *L. paracasei* over *L. plantarum*, the study next investigated whether the microbiota reshaped by *L. paracasei* alone is sufficient to mediate intestinal repair through fecal microbiota transplantation (FMT). FMT was performed with intestinal microbiota depleted recipient mice (Figure 4A). After a week of acclimation, the intestinal microbiota of 4-week-old mice (*n* = 8 per group) were depleted with a cocktail of antibiotics for four weeks, followed by two-week daily gavage of water (CK) or *L. paracasei* fecal microbiota transplantation (LFMT). Diarrhea was induced in mice by providing *S. typhimurium* in the final week. *L. paracasei*-derived microbiota, effectively attenuated the body weight loss (Figure 4B), and the damage to the intestinal mucosal surface (Figures 4C–E) of *S. typhimurium*-treated mice. Furthermore, the expressions of tight junction proteins, such as Occludin, were substantially restored (Figure 4F), and the serum levels of pro-inflammatory cytokines, including TNF-*α* and IL-1β, were significantly suppressed in *S. typhimurium*-challenged mice in the LFMT group compared with the NC group (Figure 4G).

### LFMT promotes intestinal stem cell differentiation and repairs the intestinal mucosa by modulating the composition of gut microbiota and their metabolic products

**Figure 5:**
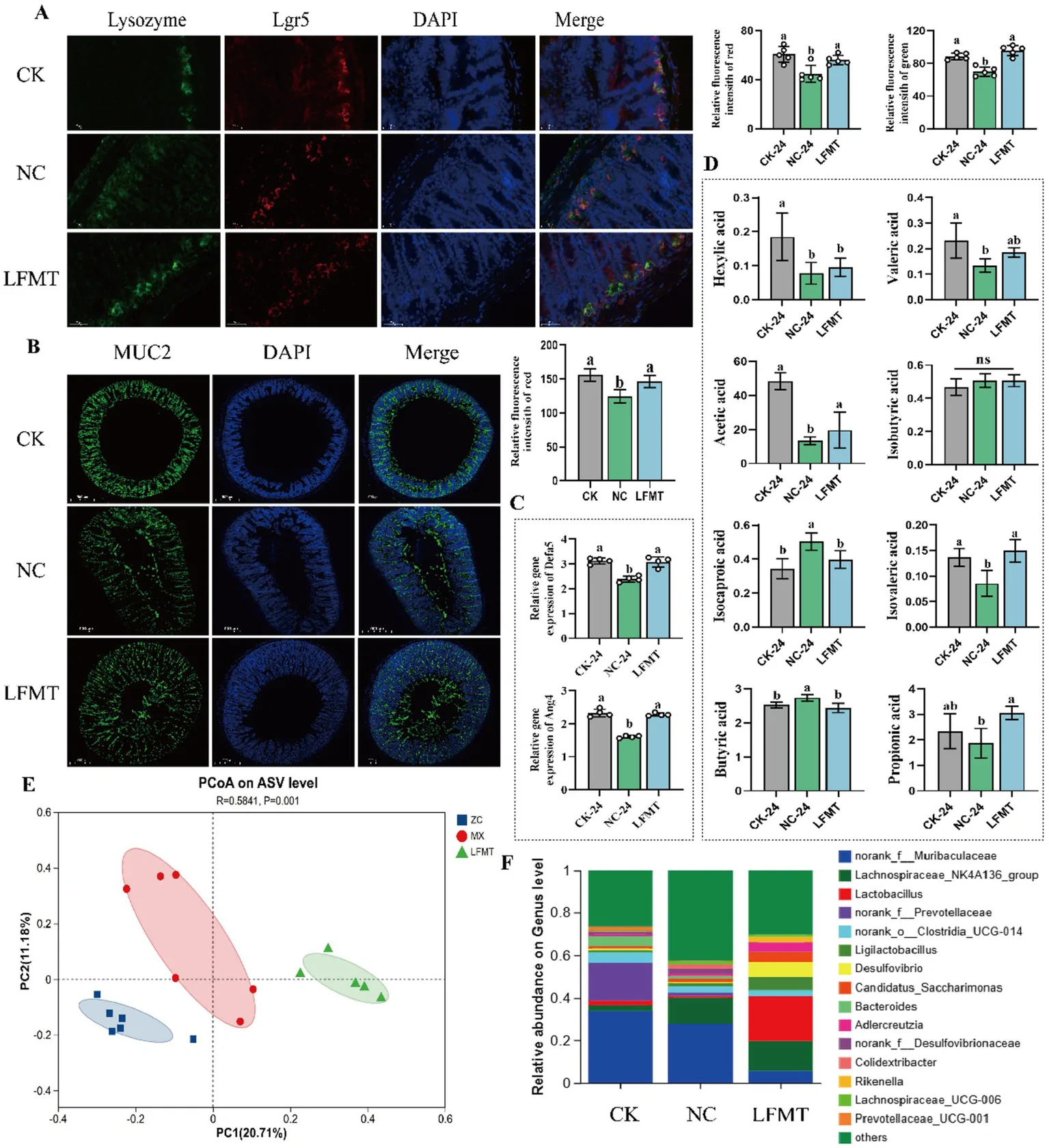
LFMT improves intestinal homeostasis by targeting the stem cell niche, mucosal barrier, microbial metabolism, and gut microbiota composition. **(A)** Representative immunofluorescence staining of lysozyme and Lgr5 in the small intestine. Right panels show semi-quantitative analysis of fluorescence intensity. **(B)** Representative immunofluorescence staining of MUC2 in the ilea. Right panel shows semi-quantitative analysis of MUC2 fluorescence intensity. **(C)** RT-qPCR analysis of relative mRNA expression levels of Defa5 and Ang4. **(D)** Concentrations of SCFAs in the ilea. **(E)** PCoA of gut microbiota based on Bray–Curtis distance. **(F)** Stacked bar plot showing the relative abundance of gut microbiota at the genus level. Data are presented as mean ± SEM. Different lowercase letters indicate significant differences between groups (*p* < 0.05); the same letter indicates no significant difference (*p* > 0.05). Ns = not significant.

To evaluate the regulation of intestinal epithelial renewal capacity by LFMT, immunofluorescence was used to detect the expression of the Paneth cell marker Lysozyme and the intestinal stem cell marker Lgr5. The results showed that compared with the CK group, the fluorescence intensities of Lysozyme and Lgr5 in the NC group were significantly downregulated, indicating impaired intestinal stem cell niche function after modeling. LFMT treatment significantly restored the expression levels of both markers, with Lgr5 fluorescence intensity returning to a level not significantly different from that of the CK group, and Lysozyme expression also significantly higher than that in the NC group, suggesting that LFMT effectively repairs the damaged intestinal stem cell niche and promotes intestinal epithelial regeneration (Figure 5A). MUC2 is a core mucin secreted by intestinal goblet cells and a key component of the intestinal mucus barrier. Immunofluorescence staining revealed that in the CK group, MUC2 protein exhibited a continuous, uniform ring-like distribution with strong and intact fluorescence signals. In the NC group, MUC2 fluorescence signals were markedly weakened, and the mucus layer structure was disrupted with interrupted continuity, indicating severe damage to the intestinal mucus barrier. Following LFMT treatment, MUC2 expression significantly recovered, the mucus layer structure tended to become intact, and the fluorescence intensity was significantly higher than that in the NC group and not significantly different from that in the CK group, demonstrating that LFMT effectively maintains the integrity of the intestinal mucus barrier and enhances the physical defense capacity of the intestine (Figure 5B). To further validate the regulatory effect of LFMT on Paneth cell function and the intestinal innate immune microenvironment, RT-qPCR was performed to assess the mRNA expression levels of key antimicrobial genes in Paneth cells (Figure 5C). Compared with the CK-24 group, the expression level of Defa5, a specific marker of Paneth cell *α*-defensins, was significantly downregulated in the NC-24 group, corroborating the reduced Lysozyme immunofluorescence signal observed in Figure 5A. Notably, LFMT treatment restored Defa5 expression to a level comparable to that of the CK-24 group. The expression of angiogenin 4 (Ang4) was also significantly lower in the NC-24 group than in the CK-24 group. LFMT intervention significantly upregulated Ang4 expression, making it significantly higher than that in the NC-24 group and not significantly different from that in the CK-24 group. These results confirm that LFMT reverses the aberrant expression of the Paneth cell effector molecules Defa5 and Ang4, thereby restoring the antibacterial function of the intestinal innate immunity and supporting the maintenance of a healthy intestinal microenvironment. SCFAs are key metabolites of the gut microbiota and play important roles in maintaining intestinal homeostasis, anti-inflammation, and regulating energy metabolism. Gas chromatography results showed that the concentrations of acetic acid, hexylic acid, valeric acid, and isovaleric acid in the intestinal contents of mice in the NC group were significantly lower than those in the CK group, indicating disturbed metabolic function of the gut microbiota under the model condition. LFMT treatment significantly elevated the concentrations of multiple SCFAs, with acetic acid, isovaleric acid, and propionic acid levels significantly higher than those in the NC group, and some indices recovered to levels not significantly different from those in the CK group. In contrast, the concentrations of isobutyric acid showed no significant differences among the three groups. These results indicate that LFMT effectively reshapes the metabolic profile of the gut microbiota, promotes the production of beneficial SCFAs, and improves the intestinal microenvironment (Figure 5D). Principal coordinate analysis (PCoA) based on Bray–Curtis distances revealed clear inter-group clustering of the gut microbiota structure among the three groups. Samples from the CK group clustered predominantly in the left region, while NC group samples clustered in the right region. LFMT group samples shifted toward the CK group and were clearly separated from NC group samples. This suggests that the modeling significantly altered the overall gut microbiota structure, and LFMT treatment effectively reversed this change, remodeling the microbiota structure toward a healthy state (Figure 5E). Compositional analysis at the genus level showed marked differences between the CK and NC groups: the relative abundances of beneficial bacteria such as *Lactobacillus* and *Lachnospiraceae_NK4A136_group* were significantly decreased in the NC group, while the abundances of some potentially harmful bacteria were increased. After LFMT treatment, the microbial composition changed notably, with the relative abundances of beneficial bacteria significantly increased, and the overall structure more closely resembling that of the CK group, further confirming the positive regulatory effect of LFMT on the gut microbiota (Figure 5F).

## Discussion

Diarrhea has emerged as a significant public health concern with rising incidences. Pathogenic microorganisms are one of the primary causes of diarrhea (22), including pathogens such as *Escherichia coli*, *Salmonella* spp., and *Staphylococcus aureus*. Following pathogenic infection, the intestinal microbiota becomes dysregulated, leading to disruption of the intestinal mucosal barrier and functional impairment (23). Moreover, pathogenic invasion compromises the proliferation of intestinal stem cells, thereby delaying the repair of damaged intestinal epithelial cells (24, 25). In this study, the protective effects of *L. paracasei* and *L. plantarum* against *S. typhimurium*-induced infectious diarrhea were investigated using a mouse model. The results demonstrate that *L. paracasei* effectively alleviated intestinal barrier dysfunction and promoted mucosal repair through a microbiota–metabolite–intestinal stem cell axis. Notably, although both strains conferred beneficial effects, *L. paracasei* exhibited significantly superior efficacy, which was associated with its unique ability to remodel the gut microbiota and enrich SCFA-producing taxa. Importantly, fecal microbiota transplantation experiments further confirmed that the microbiota reshaped by *L. paracasei* independently mediated intestinal protection.

Furthermore, the study observed that *L. paracasei* promoted goblet cell recovery and upregulated the expression of TFF3, which is a repair factor secreted by goblet cell that enhances epithelial migration and intestinal stem cell survival. These changes indicated that *L. paracasei* simultaneously strengthens the physical barrier (mucus layer) and the epithelial repair capacity. Notably, the relative abundance of bacteria belonging to the family Muribaculaceae and Prevotellaceae was markedly increased following *L. paracasei* treatment. Muribaculaceae and Prevotellaceae are known producers of SCFAs and may partially mediate the metabolic activity of the gut microbiota, thereby indirectly promoting intestinal mucosal repair. Moreover, *L. paracasei* significantly reduced serum levels of the pro-inflammatory cytokines TNF-*α*, IL-1β, and IL-6, while increasing the level of the anti-inflammatory cytokine IL-10. Together, these results suggest that *L. paracasei* can suppress systemic inflammatory, thereby creating a favorable microenvironment for the survival and expansion of ISCs. The FMT experiment further confirmed that alterations in the gut microbiota alone are sufficient to mediate anti-inflammatory effects and promote intestinal stem cell regeneration, indicating that microbial metabolites are key intermediaries. Therefore, the intestinal metabolites were further examined.

SCFA are metabolites produced by the gut microbiota through the fermentation of indigestible carbohydrates, including acetate, propionate, and butyrate, and are known to possess immunomodulatory functions (26). SCFA can promote naive T cell differentiation by modulating cellular energy status (27–29). Propionate inhibits *de novo* fatty acid synthesis by inactivating acetyl-CoA carboxylase 1, thereby promoting Treg differentiation and IL-10 production (30, 31). Studies have also shown that butyrate can induce Treg differentiation and secretory immunoglobulin A (sIgA) production through class-switch recombination under IL-10 induction (32–34). Infant formula supplemented with human milk oligosaccharides (HMO) increases IL-10 levels and fecal sIgA in infants by promoting SCFA production, indicating that the immunomodulatory effects of HMO are mediated by SCFAs (35). SCFAs not only provide energy for intestinal epithelial cells but also directly regulate ISC fate via G protein-coupled receptors and inhibition of histone deacetylases (36, 37). In this study, the enrichment of Muribaculaceae and Prevotellaceae by *L. paracasei* is of particular interest. Muribaculaceae and Prevotellaceae are recognized as key SCFA producers, and their increased abundance directly correlated with elevated fecal acetate, propionate, and butyrate levels in the *L. paracasei*-treated group. Importantly, this specific enrichment was not observed in the *L. plantarum*-treated mice. Therefore, *L. paracasei* may promote the proliferation and differentiation of intestinal stem cells in an SCFA-dependent manner.

The continuous renewal and injury repair of the intestinal epithelium are fundamentally dependent on Lgr5^+^ ISCs located at the crypt base (38, 39). The maintenance of self-renewal and lineage differentiation of Lgr5^+^ ISCs is orchestrated by multiple signaling pathways, among which the Wnt/*β*-catenin and Notch cascades play pivotal roles (39, 40). Specifically, high-intensity Notch signaling drives ISCs toward an absorptive enterocyte fate, whereas Wnt signaling promotes their differentiation into secretory lineage cells, including Paneth cells and goblet cells (41). Paneth cells constitute a critical component of the ISC niche, they reside at the crypt base in close apposition to Lgr5^+^ ISCs and support ISC proliferation and differentiation through the paracrine secretion of Wnt and Notch ligands (42, 43). In the present study, following *S. typhimurium* infection, the mRNA expression levels of lysozyme and the antimicrobial peptides Defa5 and Ang4 were markedly reduced, yet were restored to near-normal levels upon *L. paracasei* treatment, suggesting that *L. paracasei* may indirectly sustain ISC activity by preserving the functional integrity of Paneth cells. Goblet cells, as essential members of the secretory lineage, secrete MUC2 to establish the mucus barrier, and also release TFF3 to facilitate epithelial migration and wound healing (44). A reduction in goblet cell numbers and diminished MUC2 secretion during infectious diarrhea have been extensively documented (45–47). Notably, in this study, *L. paracasei* administration not only significantly restored MUC2 expression and the structural integrity of the mucus layer but also upregulated TFF3 expression, indicating that *L. paracasei* concurrently enhances physical barrier function and promotes epithelial repair capacity.

Collectively, the findings of this study demonstrate that *L. paracasei* inhibited the inflammatory response, protected Paneth cells and their supporting function to ISC by regulating the structure of intestinal flora and increasing the production of metabolites such as SCFA, and ultimately promoted the proliferation and differentiation of Lgr5^+^ ISC and repaired the damaged intestinal mucosa. This finding provides a new mechanism for the application of probiotics in infectious diarrhea, and provides a theoretical basis for the intestinal repair strategy targeting ISC. Moreover, although some studies have reported that *L. plantarum* can enrich Prevotellaceae, this study did not observe any such enrichment in the *S. typhimurium*-infected model. This discrepancy may be attributed to differences in the specific strain used, or the infection model. Nevertheless, the findings clearly indicate that the efficacy of probiotic strains in infectious diarrhea is closely linked to their specific microbiota-modulating properties, and *L. paracasei* may represent a more promising candidate for targeting the microbiota–ISC axis.

## Data Availability

The data presented in the study are deposited in the NCBI Sequence Read Archive (SRA) repository, accession number PRJNA1508840.

## Footnotes

1
www.majorbio.com
